# Characterization of the major formamidopyrimidine–DNA glycosylase homolog in *Mycobacterium tuberculosis* and its linkage to variable tandem repeats

**DOI:** 10.1111/j.1574-695X.2009.00562.x

**Published:** 2009-07

**Authors:** Ingrid Olsen, Seetha V Balasingham, Tonje Davidsen, Ephrem Debebe, Einar A Rødland, Dick van Soolingen, Kristin Kremer, Ingrun Alseth, Tone Tønjum, Patrick Brennan

**Affiliations:** 1 Centre for Molecular Biology and Neuroscience, Institute of Microbiology, University of Oslo, Oslo University Hospital (Rikshospitalet)Oslo, Norway; 2 National Veterinary InstituteOslo, Norway; 3 Institute of Microbiology, Oslo University Hospital (Rikshospitalet)Oslo, Norway; 4 Norwegian Computing CenterOslo, Norway; 5 Department of Informatics, University of OsloOslo, Norway; 6 Mycobacteria Reference Unit, National Institute of Public Health and the EnvironmentBilthoven, The Netherlands

**Keywords:** *Mycobacterium tuberculosis*, DNA repair, formamidopyrimidine–DNA glycosylase, tandem repeats

## Abstract

The ability to repair DNA damage is likely to play an important role in the survival of facultative intracellular parasites because they are exposed to high levels of reactive oxygen species and nitrogen intermediates inside phagocytes. Correcting oxidative damage in purines and pyrimidines is the primary function of the enzymes formamidopyrimidine (faPy)–DNA glycosylase (Fpg) and endonuclease VIII (Nei) of the base excision repair pathway, respectively. Four gene homologs, belonging to the *fpg/nei* family, have been identified in *Mycobacterium tuberculosis* H37Rv. The recombinant protein encoded by *M. tuberculosis Rv2924c*, termed Mtb-Fpg1, was overexpressed, purified and biochemically characterized. The enzyme removed faPy and 5-hydroxycytosine lesions, as well as 8-oxo-7,8-dihydroguanine (8oxoG) opposite to C, T and G. Mtb-Fpg1 thus exhibited substrate specificities typical for Fpg enzymes. Although *Mtb-fpg1* showed nearly complete nucleotide sequence conservation in 32 *M. tuberculosis* isolates, the region upstream of *Mtb-fpg1* in these strains contained tandem repeat motifs of variable length. A relationship between repeat length and *Mtb-fpg1* expression level was demonstrated in *M. tuberculosis* strains, indicating that an increased length of the tandem repeats positively influenced the expression levels of *Mtb-fpg1*. This is the first example of such a tandem repeat region of variable length being linked to the expression level of a bacterial gene.

## Introduction

*Mycobacterium tuberculosis* is a predominant cause of tuberculosis infections worldwide, with the highest incidence found in developing countries. This pathogen is a member of the *M. tuberculosis* complex (MTC), which contains the genetically highly conserved species *Mycobacterium bovis, Mycobacterium africanum, Mycobacterium canettii, M. bovis* BCG, *Mycobacterium caprae, Mycobacterium pinnipedii* and *Mycobacterium microti*. As a facultative intracellular pathogen, *M. tuberculosis* survives and replicates inside human macrophages. Accordingly, it resides in a hostile environment where reactive oxygen and nitrogen radicals can induce deleterious effects, including DNA damage. Consequences of DNA damage comprise single- and double-strand breaks, abasic (apurinic/apyrimidinic, AP) sites and base damages that can have cytotoxic or mutagenic effects (Bjelland & Seeberg, 2003). Thus, in order to survive, intracellular bacteria must be particularly capable of repairing oxidative and nitrosative DNA lesions.

In *Escherichia coli*, subtle base alterations in DNA of endogenous origin are primarily repaired by the base excision repair (BER) pathway (Seeberg *et al.*, 1995). The repair of oxidized bases is initiated by the activity of a DNA glycosylase belonging to the formamidopyrimidine (faPy)–DNA glycosylase (Fpg)/endonuclease VIII (Nei) family. *Escherichia coli* Fpg (Fpg Ec), encoded by the *mutM* gene, primarily catalyses excision of 8oxoG and other oxidatively damaged purines from DNA, while the principal substrates of *E. coli* Nei (Nei Ec) are oxidized pyrimidines. Although Fpg and Nei have different substrate specificities, they share common N- and C-terminal domains and a similar enzymatic mode of action with three types of activities: hydrolysis of the *N*-glycosidic bond with transient formation of an AP site (DNA glycosylase activity) and cleavage of the sugar–phosphate backbone 3′ to the abasic site (β-elimination) before the 5′-cleavage (δ-elimination) (Melamede *et al.*, 1994; Bhagwat & Gerlt, 1996; Zharkov *et al.*, 2003). Consecutive activities of these three functions remove the lesion from duplex DNA, leaving a single-nucleotide gap in the damaged strand flanked by phosphate residues.

While there is an abundance of studies addressing oxidative damage repair mechanisms in *E. coli*, reports on these aspects in mycobacteria are scarce. Based on the genome sequences of *M. tuberculosis* strains, no less than four homologs of the *fpg/nei E. coli* genes have been identified (Mizrahi & Andersen, 1998). Recently, two of these genes from *M. tuberculosis*, encoding *Mtu*-Nei2 and *Mtu*-Fpg2, were cloned and characterized (Sidorenko *et al.*, 2008). Another study on an Fpg (MutM)-deficient *Mycobacterium smegmatis* revealed a remarkable increase in the accumulation of A to G (T to C) mutations, in contrast to Fpg-deficient *E. coli*, where C to A mutations predominate (Jain *et al.*, 2007).

The genome of *M. tuberculosis* contains several kinds of repetitive DNA sequences, including insertion sequences, major polymorphic tandem repeats, polymorphic GC-rich repetitive sequences, direct repeats, variable number of tandem repeats (VNTR) and *Mycobacterium* interspersed repetitive units (MIRU) (van Soolingen *et al.*, 1993; Supply *et al.*, 1997; Smittipat & Palittapongarnpim, 2000). The VNTRs often differ in copy number between isolates (Frothingham & Meeker-O'Connell, 1998). Structures consisting of 40–100-bp repetitive sequences, termed MIRUs, have been found scattered in 41 locations in the *M. tuberculosis* H37Rv chromosome; 12 of these were polymorphic in MIRU copy numbers between isolates (Supply *et al.*, 1997, 2000; Magdalena *et al.*, 1998). The tandem repeats, predominantly MIRU-VNTR, are commonly used in genotyping of *M. tuberculosis* isolates for routine epidemiological discrimination (Supply *et al.*, 2006), and one of these, previously termed exact tandem repeat (ETR)-F and VNTR3239, has been detected upstream of *Rv2924c* (Frothingham & Meeker-O'Connell, 1998; Smittipat & Palittapongarnpim, 2000). However, the exact function of these repeats in *M. tuberculosis* is not well understood. Proposed possible functions for MIRU-VNTR include regulation of gene expression, differential translation of genes within a polycistronic operon, or some may serve as structural components for chromosome organization (Supply *et al.*, 1997).

In this study, the ORF of the main *fpg*-homolog *Rv2924c* was cloned, and its gene product, termed Mtb-Fpg1, was biochemically characterized. Recombinant Mtb-Fpg1 was assessed with regard to its enzymatic activity toward a panel of DNA substrates containing single lesions. The DNA sequences of *Mtb-fpg1* homologs and their upstream regions in *M. tuberculosis* and other mycobacterial species were compared and led to the characterization of the MIRU-VNTR tandem repeat region upstream of *Mtb-fpg1*. This tandem repeat region was characterized in a panel of *M. tuberculosis* isolates and other mycobacterial species, and the impact of their length on *Mtb-fpg1* gene expression was assessed.

## Materials and methods

### Mycobacterial genome sequences and bioinformatics tools

The genome sequences of *M. tuberculosis* H37Rv (http://genolist.pasteur.fr/TubercuList/) (Cole *et al.*, 1998), F11 (http://www.broad.mit.edu/), and CDC1551 (Accession no. NC_002755) (Fleischmann *et al.*, 2002), *M. bovis* AF2122/97 (Garnier *et al.*, 2003) (http://genolist.pasteur.fr/BoviList), *Mycobacterium leprae* TN (Cole *et al.*, 2001) (http://genolist.pasteur.fr/Leproma), *Mycobacterium avium* ssp. *paratuberculosis* K10 (Accession no. NC_002944), *M. smegmatis* mc^2^155 (Accession no. NC_008596), *M. avium* 104 (http://tigrblast.tigr.org/), *Mycobacterium ulcerans* Agy99 (http://genolist.pasteur.fr/BuruList/index.html), *Mycobacterium* sp. JLS (http://www.jgi.doe.gov/), KMS (http://www.jgi.doe.gov/), MCS (http://www.jgi.doe.gov/), *Mycobacterium vanbaalenii* PYR-1 (http://www.jgi.doe.gov/) and *Mycobacterium marinum* (Accession no. NC_010612), and the incomplete genomes of *M. tuberculosis* 210 (http://tigrblast.tigr.org/ufmg), C (accession no. NZ AAKR00000000), *M. microti* OV254 (http://www.sanger.ac.uk/Projects/M_microti/) and *Mycobacterium flavescens* (accession no. NZ AAPA00000000) were searched for the presence of *fpg/nei* homologs and, when feasible, repetitive sequences between the *rnc* and *fpg* genes. The different *fpg*/*nei* orthologs and single-repeat motifs within each *Mycobacterium* genome were identified by blast searches (http://genolist.pasteur.fr). Tandem repeats were identified using tandemrepeatsfinder (http://tandem.bu.edu/trf/trf.html) (Benson, 1999). The parameters were set to 1, 4, 4, 80, 10, 100 and 500, aiming to find tandem repeats containing near-identical repeats of some length. Overlapping tandem repeat regions were joined and regarded as one region. Operons were predicted using VIMSS Operon Prediction (http://www.microbesonline.org/) (Price *et al.*, 2005). The VIMSS database was also used to identify *fpg/nei* orthologs in bacterial species other than mycobacteria.

### Media, bacterial strains and DNA manipulations

Bacterial strains and plasmids used in this study are listed in Supporting Information, Table S1. The *M. tuberculosis* collection included a wide variety of genotypes, such as the Bejing and Haarlem genotypes, which were previously shown to have mutations in putative mutator genes (Rad *et al.*, 2003). Some of the strains were previously characterized in detail by multiple genetic markers (Kremer *et al.*, 1999, 2005; Viana-Niero *et al.*, 2001). DNA isolation, PCR amplification and cloning were performed according to standard techniques (Sambrook & Russell, 2001). DNA sequencing was conducted using an Applied Biosystems 3730 Genetic Analyser System (Applied Biosystems, Foster City, CA), ABI BigDye Terminator v. 3.1 DNA sequencing kit (Applied Biosystems, Foster City, CA) and the primers listed in Table S2. The *M. tuberculosis* H37Rv *Rv2924c* (*fpg*) gene was amplified by PCR and cloned into the expression vector pET22b (Novagen, Madison, WI) encoding a C-terminal His-tag. *Escherichia coli* ER2566 (New England Biolabs, Beverly, MA) and BK3004 (Alseth *et al.*, 1999) were transformed with the pET22b plasmids by standard methods (Sambrook & Russell, 2001). For the gene expression studies, the *M. tuberculosis* strains were selected so as to be representative for the VNTR3239 tandem repeat types (TRT) 2, 3, 4 and 6. *Mycobacterium tuberculosis* strain 29593, representing the VNTR3239 TRT 6, had IS6110 restriction fragment length polymorphism (RFLP) genotype 01403120 (similar to strain NLA009700438).

### *Mycobacterium tuberculosis* Fpg purification

*Escherichia coli* strain BK3004 (*fpg*^−^) was used for overexpressing *M. tuberculosis* Mtb-Fpg1 from the plasmid pET22b-*Rv2924c*. BK3004 was grown in Luria–Bertani broth with ampicillin (100 μg mL^−1^) at 37 °C while shaking until the OD_600 nm_ was 1.0, when 1 mM isopropyl-d-thiogalactopyranoside was added and the cells were incubated with shaking for 3 h. Cells were harvested and washed in lysis buffer (300 mM NaCl, 25 mM Tris and 10 mM imidazole, pH 8.0) before subjecting them to mechanical lysis in a French Pressure Cell (SLM Aminco, Spectronic Instruments Inc., Rochester, NY). The cleared lysate was loaded onto an Ni-NTA column (Qiagen, Hilden, Germany), washed with 300 mM NaCl, 25 mM Tris, pH 7.5, and eluted with 100, 150 and 200 mM imidazole. The purified protein was dialyzed against 300 mM NaCl and 25 mM Tris, pH 8.0, overnight.

### Assay for enzymatic cleavage of DNA substrates

Duplex DNA substrates containing a single 8oxoG opposite of C, A, G, T, 5-hydroxycytosine (5OHC): G, 5-hydroxyuracil (5OHU): G, DHU: G, U: A or U: G base pair were generated by ^32^P-5′ end-labeling of oligonucleotides using T4 polynucleotide kinase (New England Biolabs) as described previously (Eide *et al.*, 1996). The oligonucleotide sequences of the DNA substrates used are listed in Table S2. DNA glycosylase reactions were performed by mixing purified protein as indicated, with 10–50 fmol DNA substrate in a total volume of 15 μL. The enzyme activities were assayed in a reaction buffer containing 70 mM 3-(*N*-morpholino) propane sulfonic acid, pH 7.5, 1 mM EDTA, 1 mM dithiothreitol and 5% glycerol, and incubated at 37 °C for 1 h. Fpg Ec (New England Biolabs) was included as the positive control. Products of the reactions were separated by 20% denaturing polyacrylamide gel electrophoresis (PAGE) and visualized by phosphorimaging.

### Assessment of alkylbase and faPy–DNA glycosylase activities

Alkylated DNA and met-faPy substrates were prepared as described previously, using *N*-[H^3^]-methyl-*N*′-nitro sourea (1.5 Ci mmol^−1^) and calf thymus DNA (6000 d.p.m. μg^−1^ DNA) (Riazuddin & Lindahl, 1978) or poly(dG–dC) (12 000 d.p.m. μg^−1^) (Boiteux *et al.*, 1984), respectively. Removal of bases was measured in a total reaction volume of 50 μL containing 7 μg DNA substrate and enzyme as indicated.

### Molecular strain typing

The recommended international standard protocol for IS*6110*-based RFLP typing was performed (Kamerbeek *et al.*, 1997). The IS*6110*-based RFLP patterns were analyzed using bionumerics software (Applied Maths, Sint-Martems-Latem, Belgium). Similarities between RFLP patterns were calculated using the Dice coefficient, and the dendrogram was prepared with the unweighted pair group method using arithmetic averages algorithm.

### RNA isolation

*Mycobacterium tuberculosis* strains were grown in Middlebrook 7H9 medium with ADC and 0.05% Tween 80 at 37 °C while shaking until OD_600 nm_ was 0.5. For the quantitative reverse transcriptase (RT)-PCR, bacterial cells were collected in tubes containing RNA-later, harvested by centrifugation and resuspended in Trizol (Invitrogen, Carlsbad, CA). Cell suspensions were transferred to FastRNA BLUE Tubes (BIO 101 Inc., La Jolla, CA) and processed in a MagNA Lyser (Roche Applied Science, Penzberg, Germany). Chloroform was then added to the cell lysates and mixed well. The tubes were centrifuged and supernatants were transferred to microcentrifuge tubes containing an equal volume of ethanol. After mixing, the solutions were applied to RNeasy spin columns (Qiagen) and treated according to the manufacturer's protocol. Total RNA was eluted with RNAse-free water and DNA was removed with TURBO DNA-*free*™ (Ambion, Hurtingdon, UK). The amount of total RNA was quantified using NanoDrop (ND-1000, NanoDrop Technologies, Wilmington, DE). All RNA isolations were performed, under cold conditions when required, on at least three independent cultures for each strain studied.

### Quantitative RT-PCR

For quantitative real-time RT-PCR, *M. tuberculosis* strains representing TRT 2, NLA000100560 (2-1) and NLA009801353 (2-2); TRT 3, NLA009802122 (3-2) and H37Rv (3-3); TRT 4, NLA009700438 (4); and TRT 6, 29593 (6) were grown, and RNA was isolated as described above. cDNA was synthesized from isolated total RNA using high-capacity cDNA reverse transcription kit (Applied Biosystems, Foster City, CA) according to the manufacturer's protocol.

Primers for *Mtb-fpg1, nei2* and the housekeeping gene *sigA* were designed to obtain a product of similar size and the same optimal annealing temperature. The Power SYBR® Green PCR master mix (Applied Biosystems, Warrington, UK) and StepOnePlus™ instrument (Applied Biosystems, Foster City, CA) were used in the two-step RT-PCR. The quantitative PCR reaction was performed in quadruplicates on cDNAs with specific primers under the following conditions: 10 min hold at 95 °C and 40 cycles of 95 °C for 15 s and 60 °C for 60 s, and then melting curve analysis was performed at 95 °C for 15 s, 60 °C for 60 s and 95 °C for 15 s. Negative controls, consisting of no-template (water) and RNA in the reaction mixtures, were run with all reactions to test for DNA contamination. The experiments were repeated at least six times. PCR products were also run on agarose gels to confirm the formation of a single product of the desired size. For the relative quantification of mRNA, the comparative *C*_t_ method (ΔΔ*C*_t_) was used to calculate the relative expression levels in each strain, normalized to *sigA* (endogenous control) and then given as fold change relative to *Mtb-fpg1* and *nei2* gene expression of *M. tuberculosis* H37Rv (calibrator). The two-sample *t*-test was performed to check for statistically significant differences in *Mtb-fpg1* and *nei2* expression level between *M. tuberculosis* strains.

## Results

### Mycobacterial genes with homology to *E. coli fpg* and *nei*

The *fpg/nei* orthologs of *M. tuberculosis* H37Rv, *M. bovis* AF2122/9, *M. leprae* TN, *M. a. paratuberculosis* K10, *M. avium* 104 and *M. smegmatis* mc^2^155 were identified and presented in Table 1. Four putative ORFs with homology to the *fpg/nei* family are present in the genome sequence of *M. tuberculosis* H37Rv: *Rv0944* (*Mtu-fpg2*), *Rv2464c* (*Mtu-nei2*), *Rv2924c* (*Mtb-fpg1*) and *Rv3297* (putative *nei*) (Cole *et al.*, 1998; Sidorenko *et al.*, 2008). The amino acid sequences exhibited significant similarities to characteristic motifs and conserved residues of the bacterial Fpg/Nei family, including a DNA glycosylase catalytic domain and a helix–two-turns–helix DNA-binding motif (Fig. S1). The catalytic residues at the N-terminus were conserved in Rv2464c, Mtb-Fpg1 and Rv3297, while Rv0944 encoded a truncated protein lacking the N-terminal part. Highly conserved proteins with 100% amino acid identity to all these four DNA glycosylases are also present in the *M. bovis* genome sequences (Table 1). In contrast, only a single complete *fpg* ortholog of *Mtb-fpg1* is present in the *M. leprae* genome (80% identity and 88% similarity at the deduced amino acid level). *Mycobacterium leprae* orthologs to *Rv0944* and *Rv2464c* are, however, present as pseudogenes, which are abundant in the *M. leprae* genome (Cole *et al.*, 2001). The *M. smegmatis* genome also harbors orthologs to the four *fpg/nei* genes with amino acid similarities ranging from 75% to 82%. In *M. smegmatis*, however, the *Rv0944c* ortholog is complete. The *M. avium* and *M. a. paratuberculosis* genome sequences contain all four *fpg/nei* orthologs exhibiting amino acid similarities of 85% to 90%, including a full-length *Rv0944* ortholog. Surprisingly, a fifth *fpg/nei* homolog whose predicted gene product contains all the conserved domains of enzymes belonging to the Fpg/Nei family was identified in the genome of *M. a*. ssp. *paratuberculosis* K10.

**Table 1:** Fpg/Nei DNA glycosylase orthologs present in the order *Actinomycetales*, including mycobacterial species

	Fpg/Nei orthologs				
Species	Putative Fpg	Putative Fpg	Putative Nei	Putative Nei	Putative Fpg/Nei?
*M. leprae* TN	ML1658	–	–	–	–
*M. tuberculosis* CDC1551	MT2994	MT0970	MT2539	MT3396	–
*M. tuberculosis* H37Rv	Rv2924c*	Rv0944	Rv2464c	Rv3297	–
*M. bovis* AF2122/97	Mb2949c	Mb0969	Mb2491C	Mb3325	–
*M. marinum*	MM1783	MM4559	MM3812	MM1237	–
*M. ulcerans*	MUL2031	MUL3737	MUL2650	MUL4418	–
*M. avium* 104	MAV3782	MAV3149	MAV1066	MAV1708	–
*M. avium* ssp. *paratuberculosis* K10	MAP2994c	MAP0889	MAP2284c	MAP3416	MAP1328c
*M. smegmatis* mc^2^ 155	SMEG2419	SMEG4683	SMEG5545	SMEG1756	–
*Streptomyces avermitilis* MA-4680	SAV2664	SAV7289	SAV2501	SAV5427	–
*Streptomyces coelicolor* A3(2)	SCO0945	SCO5573	SCO2626	SCO5760	–
*Corynebacterium efficiens* YS-314	CE1975	CE2834	CE0922	–	–
*Corynebacterium glutamicum*	NCg10813	NCg11993	NCg12898	–	–
*Corynebacterium diphtheriae*	DIP1543	DIP0829	DIP2304	–	–
*Leifsonia xyli* ssp. *xyli* str. CTCB07	Lxx00140	Lxx09800	Lxx20840	Lxx08780	–
*Nocardia farcinica*	Nfa13280	Nfa41830	NFA50200	NFA9770	–
*Propionibacterium acnes* KPA171202	PPA1451	PPA1623	–	–	–
*Tropheryma whippeli* TW08/27	TW374	–	–	–	–
*Tropheryma whippeli* str. *Twist*	TW396	–	–	–	–
*Bifidobacterium longum* NCC2705	–	–	–	–	–
*E. coli*†	MutM	–	Nei	–	–

* Now termed Mtb-Fpg1.

† Included as a reference strain.

–, none.

### Biochemical characterization of *M. tuberculosis* Mtb-Fpg1

**Fig. 1:**
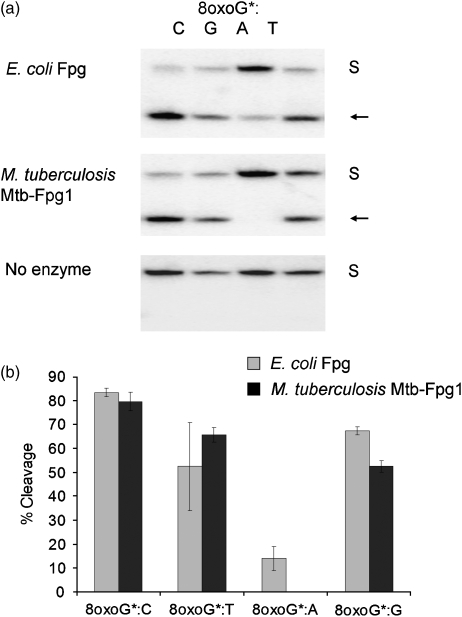
DNA glycosylase activity of *Mycobacterium tuberculosis* Mtb-Fpg1. (a) An aliquot of 50 ng of purified Mtb-Fpg1 or Fpg Ec was incubated with 10–50 fmol of a 24-bp duplex oligodeoxyribonucleotide containing a single 8oxoG residue opposite G, C, A or T. Base excision and strand cleavage were analyzed by 20% PAGE and phosphorimaging. The arrow indicates the cleaved DNA substrate. ^*^P^32^-labeled strand. S, substrate. (b) Quantification of strand cleavage activity. The results represent the averages of three independent experiments and error bars indicate the SE of the mean.

**Fig. 2:**
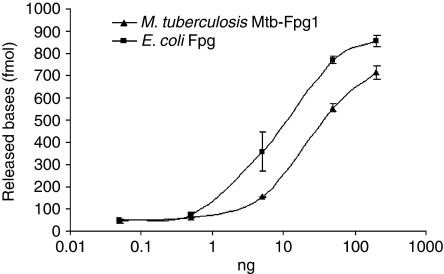
FaPy–DNA glycosylase activity of *Mycobacterium tuberculosis* Mtb-Fpg1. Removal of met-faPy from [3H]-methyl-faPy-poly(dG–dC) DNA by increasing amounts of purified Mtb*-*Fpg1 (triangles) and Fpg Ec (squares). Results represent the averages of three independent experiments and error bars indicate SE of the mean.

The *Mtb-fpg1*-encoded protein was purified to homogeneity and found to migrate at *c*. 32 kDa in sodium dodecyl sulfate-PAGE, corresponding to the molecular weight predicted from the genome sequence (31.95 kDa) (data not shown). The ability of recombinant *M. tuberculosis* Mtb-Fpg1 to remove an 8oxoG lesion was compared with that of Fpg Ec. Mtb-Fpg1 was able to remove 8oxoG when paired with C, G and T, while no activity was detected against 8oxoG:A (Fig. 1). In addition to DNA glycosylase activity, recombinant Mtb-Fpg1 protein also possessed strand cleavage activity (Fig. 1a). Mtb-Fpg1 has not been tested previously for activity toward substrates other than 8oxoG. Another typical substrate for Fpg protein is faPy residues. The *M. tuberculosis* Mtb-Fpg1 protein was tested for the ability to remove met-faPy lesions in DNA, and a similar capacity for Mtb-Fpg1 to excise met-faPy residues was observed as for Fpg Ec (Fig. 2). The recombinant Mtb-Fpg1 protein was further assessed for its ability to remove oxidized pyrimidines, 5OHC, 5OHU and diHU, in addition to uracil, alkylated bases and adenine opposite 8oxoG. Some cleavage activity was detected toward 5OHC, whereas no activity was present toward the other substrates (Table S3). In conclusion, these results demonstrate that the protein encoded by *M. tuberculosis Mtb-fpg1* is an Fpg DNA glycosylase.

### Characterization of the *Mtb-fpg1* and *rnc* intergenic region in mycobacterial species with MIRU-VNTR repeats

The sequences of the *Mtb-fpg1* gene and flanking regions were compared in a panel of mycobacterial species for which the complete genome sequences were available. The *rnc, Rv2927c* and *Rv2926c* genes were present in all the mycobacterial species examined, and their location and deduced amino acid sequences were highly conserved (data not shown). However, the intergenic region between *rnc* and *Mtb*-*fpg1* revealed significant differences in the number of base pairs. The corresponding intergenic region in *M. tuberculosis* (strains H37Rv, CDC1551, 210, C and F11), *M. bovis* and *M. microti* contained 358, 303 and 279 bp (Table 2), respectively, and these regions consisted of a complex organization of multiple direct repeats. In contrast, in the non-MTC members, the corresponding intergenic region contained only a few base pairs from the stop codon of *rnc* to the start codon of the *Mtb-fpg1* orthologs (Table 2).

**Table 2:** Mycobacterial species with MIRU-VNTR repeats

*Mycobacterium* species	Intergenic region *rnc-Mtb-fpg1* (bp)
*M. tuberculosis* H37Rv*	358
*M. tuberculosis* CDC1551*	358
*M. tuberculosis* 210†	358
*M. tuberculosis* C†	358
*M. tuberculosis* F11*	358
*M. bovis* AF2122/97*	303
*M. microti* OV254†	279

*M. leprae* TN*	54
*M. a. paratuberculosis* K10*	20
*M. avium* 104*	20
*M. smegmatis* mc^2^ 155*	6
*M*. sp. JLS*	6
*M*. sp. KMS*	6
*M*. sp. MCS*	6
*M. vanbaalenii* PYR-1*	6
*M. flavescens* PYR-GCK†‡	6
*M. marinum* (M strain)*	3
*M. ulcerans* Agy99*	1

* Completely sequenced genome.

† Incompletely sequenced genome.

‡ Sequence homology to *Mycobacterium* sp. JLS, *Mycobacterium* sp. KMS and *Mycobacterium* sp. MCS, as well as *Mycobacterium vanbaalenii* indicate no more than 6 bp from the stop codon of *rnc* to the start codon of *fpg*.

Species belonging to the *Mycobacterium tuberculosis* complex (above the line) exhibit long intergenic regions between *rnc* and *Mtb-fpg1* genes, contrasting those in non-*M. tuberculosis* complex species (below the line). The length of the intergenic region is a marker for the presence of repeats.

**Fig. 3:**
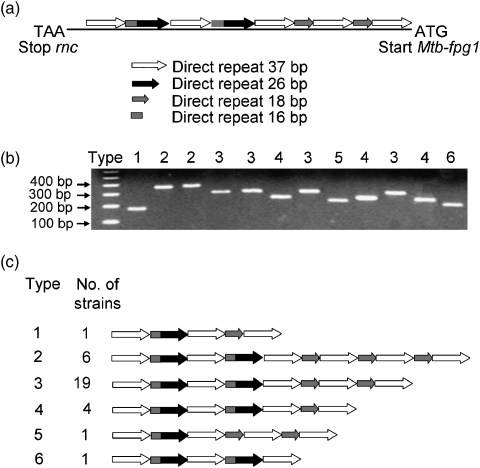
The *Mycobacterium tuberculosis* Mtb-fpg1 region contains variable numbers of repeat units. (a) Organization of the repetitive elements in *M. tuberculosis* H37Rv. The start of the repetitive structure is located 40 bp after the stop codon of *rnc* and ends at the start codon of *Mtb-fpg1*. Arrows indicate length of the repeats. In direct repeat 18 bp, 16 out of 18 consecutive base pairs are identical to direct repeat 16 bp. (b) Variation in the *rnc-Mtb-fpg1* intergenic region in *M. tuberculosis* isolates monitored by PCR amplification. Number indicates PCR product size/repeat type according to (c). (c) The organization of the different types of *M. tuberculosis Mtb-fpg1* tandem repeat regions found in 32 *M. tuberculosis* strains. Arrows as in (a).

The intergenic region between *Mtb*-*fpg1* and *rnc* in *M. tuberculosis* H37Rv described above consisted of a complex structure of exact, direct repeats (Fig. 3a). This structure contained five identical copies of a 37-bp repeat, where the last two repeats were preceded by a smaller 18-bp repeat. The second and third repeats were both preceded by a 26-bp repeat element and a 16-bp element that was identical to the 5′ end of the 18-bp repeat (Fig. 3a). The attenuated *M. tuberculosis* H37Ra is predicted to have an ORF encoding a 63 amino acid long hypothetical protein, MRA_2951, within this intergenic region (Zheng *et al.*, 2008). Even though the identical DNA sequence is found in other completed *M. tuberculosis* genome sequences, this ORF is not annotated in those. Furthermore, the complete genome sequence of *M. tuberculosis* H37Ra is not yet validated, weakening the significance of the putative ORF MRA_2951. To assess whether this repeat organization was representative for that in other *M. tuberculosis* isolates, the corresponding regions upstream of *Mtb-fpg1*, in addition to the *Mtb-fpg1* genes themselves, from 32 clinical isolates were subjected to PCR and DNA sequence analysis. The results showed that the *Mtb-fpg1* gene was almost completely conserved, and only a single point mutation, inducing an amino acid change in a region flanking an active domain, was found in a *M. tuberculosis* isolate (NLA000301029). In contrast, a total of six different classes of repeat organization patterns upstream of *Mtb-fpg1* were observed among these isolates (Fig. 3b). The most predominant variant, termed type 3, was identical to the repeat structure found in *M. tuberculosis* H37Rv, while the type 4 was the variant also found in *M. bovis* AF2122/97. The structures of the various repeats are shown in Fig. 3c.

### Correlation of *M. tuberculosis* strain typing and repeat occurrence

**Fig. 4:**
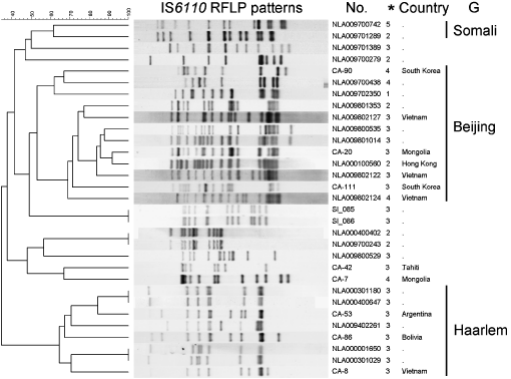
IS6110 RFLP patterns of the *Mycobacterium tuberculosis* strains. IS*6110* RFLP patterns of the *M. tuberculosis* strains used in this study. ^*^rnc-Mtb-fpg1 repeat type; G, genotype.

IS*6110* RFLP typing of 31 selected *M. tuberculosis* strains, originating from various parts of the world, showed a high degree of DNA polymorphism among the isolates (Fig. 4). Eight RFLP patterns were clustered into four groups of two identical patterns each. These isolates represented microepidemics of tuberculosis in the Netherlands. In order to investigate possible differences between nonclustered, endogenous reactivation cases (from which no transmission had occurred) and clustered cases, isolates representing each of the two identical patterns were also included in the collection (Table S1). However, no clear correlation was observed between the *rnc-Mtb-fpg1* TRT and IS*6110* RFLP type, genotype or level of success among the strains. In isolates of the Somali and Beijing genotype, various *rnc-Mtb-fpg1* TRTs were observed. For instance, the Beijing strains collectively exhibited TRTs designated 1, 2, 3 and 4, although TRT 3 was generally most abundant. In contrast, for the isolates belonging to other IS*6110* RFLP clusters and Haarlem genotype, identical *rnc-Mtb-fpg1* TRTs, were observed within each of these groups, indicating that these repeats evolve more slowly than IS*6110*, or that other repeat variants are selected against.

### The relationship between tandem repeat length and *Mtb-fpg1* expression levels

The four ORFs, *Rv2927c* (hypothetical protein), *Rv2926c* (hypothetical protein), *rnc* and *Mtb-fpg1*, were located on the same strand, and they were predicted to be part of the same operon in *M. a. paratuberculosis* and *M. leprae* using VIMSS Operon Prediction. This algorithm might, however, not be ideal for mycobacterial operon prediction. In *M. tuberculosis* species, due to the long distance between *rnc* and *Mtb-fpg1*, only *Rv2927c, Rv2926c* and *rnc* were predicted to be in the same operon.

**Fig. 5:**
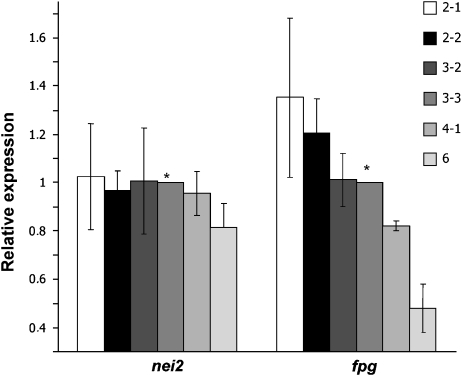
Expression analysis of *Mycobacterium tuberculosis Mtb-fpg1*. Analysis of the relative expression of *Mtb-fpg1 and nei2* (*Rv2464c*) mRNA in *M. tuberculosis* strains containing different TRTs upstream of the *Mtb*-*fpg1*. Data are expressed as fold change relative to *M. tuberculosis* H37Rv *Mtb-fpg1* or *nei2* expression when normalized to *sigA*. Data represent the average of six independent experiments. Error bars indicate SE of the mean. TRT as in Fig. 3c. Strains: 2-1, NLA0001000560; 2-2, NLA009801353; 3-2, NLA009802122; 3-3, H37Rv; 4, NLA 009700438; 6, 29593. ^*^Calibrator.

The transcription levels of *M. tuberculosis Mtb-fpg1* was assessed using quantitative real time RT-PCR in representative *M. tuberculosis* strains containing different TRTs (Fig. 5). The transcription level of *Mtb-fpg1* mRNA was compared with the transcription level of *nei2* mRNA. While the transcription of *nei2* mRNA level was more or less the same in the strains examined (*P*>0.05), the level of *Mtb-fpg1* transcription decreased with decreasing intergenic repeat length (Fig. 5). An increase in *Mtb-fpg1* expression level was observed in strains NLA000100560 (2-1) (*P*<0.05) and NLA009801353 (2-2) (*P*<0.05), which contain the longest repeat, as compared with H37Rv, while strain NLA009802122 (3-2) (*P*>0.05) exhibited an *Mtb-fpg1* expression level equal to H37Rv (3-3). Strain NLA009700438 (4) (*P*<0.05) showed lower *Mtb-fpg1* expression level than H37Rv. Strain 29593 (6) (*P*<0.05) had the lowest expression level of *Mtb-fpg1*. Even though the fold changes in the *Mtb-fpg1* expression levels were modest among the strains tested, the differences between the changes observed were statistically significant. In general, the longer tandem repeat regions within a strain in our experiments were correlated with higher mRNA levels of *Mtb-fpg1*. Taken together, the results indicate that the length of the tandem repeat units influences *Mtb-Fpg1* expression levels.

## Discussion

The *M. tuberculosis* genome sequence has revealed the presence of putative gene homologs encoding proteins involved in BER, nucleotide excision repair, recombinational repair and SOS-induced DNA damage response (Cole *et al.*, 1998, 2001; Mizrahi & Andersen, 1998). However, no *M. tuberculosis* gene homologs encoding mismatch repair components have been recognized so far. Notably, only a few *M. tuberculosis* DNA repair components have been characterized to date. However, the lifestyle and importance of this pathogen, in particular its ability to replicate or stay dormant and survive within human macrophages, warrant the elucidation of the role of DNA repair in *M. tuberculosis*. Here, we report the characterization of *M. tuberculosis* Mtb-Fpg1, a DNA glycosylase shown to be involved in the defence against oxidative DNA damage. In addition, the repeat sequence motif immediately upstream of *Mtb-fpg1* was characterized, and genome sequence analyses demonstrated that this particular sequence motif was unique to species belonging to MTC. Gene expression analysis was used to assess whether there was a link between the tandem repeat length and *Mtb-fpg1* expression levels, and a potential correlation was observed. This finding suggests that the rnc-*Mtb-fpg1* tandem repeat units may be adaptive and could potentially affect the regulation of *M. tuberculosis* oxidative DNA damage repair.

Multiple genes encoding DNA glycosylases belonging to the Fpg/Nei family are present in MTC and the *M. avium* complex (Table 1), suggesting that the repair of oxidative DNA lesions is well developed in these mycobacteria. Alternatively, these components might exert different functions in genome maintenance. The highly human-adapted *M. leprae* genome, which has undergone major reductive evolution, has retained a single conserved *fpg* gene, indicating that the Fpg family of enzymes plays a central role in this pathogen. The presence of multiple *fpg/nei* homologs in other mycobacteria, on the other hand, suggests a need for versatile mechanisms to remove oxidized bases, and may indicate that the various *fpg* gene copies might be differentially expressed under variable conditions. Despite the limited data available on gene expression profiles of Fpg/Nei DNA glycosylases, it has been shown previously that *M. tuberculosis* Rv3297 (Nei) is induced by DNA damage by a RecA-independent pathway (Rand *et al.*, 2003). None of the four *M. tuberculosis* putative Fpg/Nei homologs have been shown to be essential for optimal growth (Sassetti *et al.*, 2003). Moreover, a study of *M. tuberculosis* transposon mutants did not exhibit reduced virulence of H37Rv *Mtb-fpg1* mutants in severe combined immunodeficiency mice (McAdam *et al.*, 2002). However, none of these studies were conducted with the entire mycobacterial Fpg/Nei family inactivated, and, thus, the significance of mycobacterial Fpg/Nei family components in fitness for survival and pathogenesis remains unknown. From both prokaryotic and eukaryotic organisms, it is known that there are multiple back-up systems among DNA glycosylases, to the extent that a single null mutant might not exhibit a clear phenotype.

Mtb-Fpg1 has been annotated as an Fpg homolog due to the presence of conserved domains and residues in the deduced amino acid sequence (Cole *et al.*, 1998). We have demonstrated that purified Mtb-Fpg1 possesses both DNA glycosylase and strand-nicking activities on representative Fpg substrates. Our results corroborate with recent characterization of *M. tuberculosis* and *M. smegmatis* Fpg/Nei orthologs (Table S4). Recently, Jain *et al.* (2007) showed that an *M. smegmatis* mutant lacking the Mtb-Fpg1 ortholog is devoid of 8oxoG repair in cell-free extracts and that this mutant is more sensitive to H_2_O_2_ than the wild type. Both 8oxoG repair and survival can be restored by complementing the mutant with a plasmid containing *M. tuberculosis Mtb-fpg1* (Jain *et al.*, 2007). Mtb-Fpg1 is thus a distinct part of the defence system against oxidative DNA damage in *M. tuberculosis*.

The MTC genomes are genetically highly conserved and variation seems to be more frequently caused by insertions and deletions rather than by base substitutions (Zheng *et al.*, 2008). Furthermore, a large amount of variation between and within the genomes of *Mycobacterium* species is due to the presence of repetitive sequence elements (Cole *et al.*, 1998; Supply *et al.*, 2000). Here, we show that the *Mtb-fpg1* gene itself was almost completely conserved among *M. tuberculosis* isolates, while polymorphisms in the tandem repeat sequence immediately upstream of *Mtb-fpg1* were identified. Organized repetitive sequence elements showing polymorphisms consisting of different numbers of tandem repeats are quite common in the *M. tuberculosis* genome and have been referred to as VNTRs, MIRUs or ETRs (Frothingham & Meeker-O'Connell, 1998; Smittipat & Palittapongarnpim, 2000; Supply *et al.*, 2000; Spurgiesz *et al.*, 2003). The tandem repeat units in the *rnc-Mtb*-*fpg1* intergenic region characterized here have previously been designated as ETR-F and VNTR3239 and have been used in *M. tuberculosis* strain typing (Frothingham & Meeker-O'Connell, 1998; Smittipat & Palittapongarnpim, 2000; Supply *et al.*, 2000; Spurgiesz *et al.*, 2003). This VNTR3239 is more complex than other VNTRs and is composed of 55-bp (37-bp+18-bp) and 79-bp (37-bp+26-bp+16-bp) repeat units of tandem repeats (Fig. 3a), while other VNTRs of *M. tuberculosis* are usually simple repeats of a single kind of sequence (Smittipat & Palittapongarnpim, 2000; Smittipat *et al.*, 2005). It has been shown that this locus exhibited variation in Beijing strains (Spurgiesz *et al.*, 2003), corroborating with our results. These repeats may be predicted to contribute to mycobacterial genome dynamics. Potentially, different tandem repeat constellations might affect DNA folding and consequent affinity, binding and interactions of transcription factors in a diversified manner, thereby affecting gene expression. Alternatively, the tandem repeats themselves might act as an enhancer by containing a variable number of binding sites for a regulator, as is the case, for example, in the regulation of serotonin transporter gene expression in brain disorders (Haddley *et al.*, 2008). Our data showed that the *Mtb-fpg1* gene was indeed differentially expressed in *M. tuberculosis* strains with long and short tandem repeat regions under standard cultivation condition. Further studies on the influence of tandem repeats on the expression of this and other genes will extend the knowledge about the function of these repeats in *M. tuberculosis* genome dynamics. Finally, the multiplicity of mycobacterial Fpg/Nei homologs and their relative role(s) in oxidative DNA damage repair awaits clarification, in order to elucidate the individual and collective physiological contributions of these homologs in intracellular survival and virulence under oxidative stress.

## Supporting Information

**Fig. S1.** Predicted domains and motifs in Fpg/Nei homologs.

**Table S1.** Bacterial strains and plasmids included in the study.

**Table S2.** DNA sequences of oligonucleotides employed in the study.

**Table S3.** Substrate specificity of *Mycobacterium tuberculosis* Mtb-Fpg1.

**Table S4.** Overview of characterization of mycobacterial Fpg/Nei orthologs to date.
