# Supplementary material for: Characterization of the major formamidopyrimidine–DNA glycosylase homolog in Mycobacterium tuberculosis and its linkage to variable tandem repeats
Source: FEMS Immunol Med Microbiol. 2009 Jul;56(2):151–61. doi: 10.1111/j.1574-695X.2009.00562.x (PMC2734929; doi:10.1111/j.1574-695X.2009.00562.x)
Supplement: Supplementary file 1 [file fim0056-0151-SD1.doc]

Supporting Information

**Characterization of the major formamidopyrimidine-DNA glycosylase homolog in *Mycobacterium tuberculosis* and its linkage to variable tandem repeats**

Ingrid Olsen^1, 2‡^, Seetha V. Balasingham^1,3‡^, Tonje Davidsen^1,3^, Ephrem Debebe^1^, Einar A. Rødland^4,5^, Dick van Soolingen^6^, Kristin Kremer^6^, Ingrun Alseth^1,3^ & Tone Tønjum^1,3^

^1^Centre for Molecular Biology and Neuroscience and Institute of Microbiology, University of Oslo, Norway, ^2^National Veterinary Institute, Oslo, Norway, ^3^Institute of Microbiology, Oslo University Hospital (Rikshospitalet), Oslo, Norway, ^4^Norwegian Computing Center, Oslo, Norway, ^5^Department of Informatics, University of Oslo, Norway , ^6^Mycobacteria Reference Unit, National Institute of Public Health and the Environment, Bilthoven, The Netherlands

**^‡^** These authors contributed equally

**Table S1. Bacterial strains and plasmids included in this study.**

| **Plasmid, strains** | **Relevant characteristic** | **Repeat type** | **Source /**  **Reference** |
| --- | --- | --- | --- |
| **Plasmids** |  |  |  |
| pET22b | Expression vector, T7 promoter-driven system, Histag, apm^R^ | - | Novagen |
| pET22b-*Rv0944* | pET22b harbouring *M. tuberculosis* *Rv0944* | - | This study |
| pET22b-*Rv2464c* | pET22b harbouring *M. tuberculosisRv2464c* | - | This study |
| pET22b-*Rv2924c* *fpg* | pET22b harbouring *M. tuberculosis Mtb-fpg1* | - | This study |
| pET22b-*Rv3297 nei* | pET22b harbouring *M. tuberculosis Rv3297 nei* | - | This study |
| **Strains** |  |  |  |
| ***Escherichia coli*** |  |  |  |
| ER2566 | Expression host with chromosomal copy of the T7 RNA polymerase gene | - | New England Biolabs |
| BL21 | Expression host with chromosomal copy of the T7 RNA polymerase gene | - | Stratagene |
| BK3004 | BL21 *fpg::kan* | - | (Alse*th et a*l., 1999) |
|  |  |  |  |
| ***Mycobacterium smegmatis*** | mc^2^155 | - |  |
| ***Mycobacterium tuberculosis*** |  |  |  |
| H37Rv |  | 3 |  |
| NLA009700438 | Beijing genotype, The Netherlands | 4 | * |
| NLA009702350 | Beijing genotype, The Netherlands | 1 | * |
| NLA009800535 | Beijing genotype, old Chinese person, The Netherlands | 3 | * |
| NLA009801014 | Beijing genotype, old Chinese person, The Netherlands | 3 | * |
| NLA009801353 | Beijing genotype, old Chinese person, The Netherlands | 2 | * |
| NLA009802122 | Beijing genotype, Vietnam | 3 | * |
| NLA009802124 | Beijing genotype, Vietnam | 4 | * |
| NLA009802127 | Beijing genotype, Vietnam | 3 | * |
| NLA000100560 | Beijing genotype, Hong Kong | 2 | * |
| CA-20 | Beijing genotype, Mongolia | 3 | * |
| CA-90 | Beijing genotype, South Korea | 4 | * |
| CA-111 | Beijing genotype, South Korea | 3 | * |
| CA-8 | Haarlem genotype, Vietnam | 3 | * |
| CA-53 | Haarlem genotype, Argentina | 3 | * |
| CA-86 | Haarlem genotype, Bolivia | 3 | * |
| NLA000001650 | Haarlem genotype, micro-epidemic cluster 73, The Netherlands | 3 | * |
| NLA000301029 | Haarlem genotype, micro-epidemic cluster 73, The Netherlands | 3 | * |
| NLA000301180 | Haarlem genotype, micro-epidemic cluster 509, The Netherlands | 3 | * |
| NLA000400647 | Haarlem genotype, micro-epidemic cluster 509, The Netherlands | 3 | * |
| NLA009700243 | Micro-epidemic cluster 510, The Netherlands | 2 | * |
| NLA000400402 | Micro-epidemic cluster 510, The Netherlands | 2 | * |
| SI-085 | Micro-epidemic cluster 103, The Netherlands | 3 | * |
| SI-086 | Micro-epidemic cluster 103, The Netherlands | 3 | * |
| NLA009402261 | Isolate from old Dutch person, The Netherlands | 3 | * |
| NLA009800529 | Isolate from old Dutch person, The Netherlands | 3 | * |
| NLA009700742 | Somali genotype, The Netherlands | 5 | * |
| NLA009701289 | Somali genotype, The Netherlands | 2 | * |
| NLA009700279 | The Netherlands | 2 | * |
| NLA009701389 | The Netherlands | 3 | * |
| CA-7 | Mongolia | 4 | * |
| CA-42 | Tahiti | 3 | * |
| 29593 | Morocco(?) | 6 |  |

^*^Source: National Institute of Public Health and the Environment (RIVM), Bilthoven, The Netherlands

**Table** S2. DNA sequences of oligonucleotides employed in the study.

| **Oligonucleotide** | **Sequence (5’-3’)** | **Purpose** | **Source** |
| --- | --- | --- | --- |
| **PCR/sequencing** |  |  |  |
| IO21 | TTTTTTTTCATATGCCCGAGCTGCCCGAAGT | Cloning of *Mtb-fpg1* | This study |
| IO22 | TTTCTCGAGCTTTCGGGGCCGCGGCTG | Cloning of *Mtb-fpg1* | This study |
| 7593 | GCAACATCTATGCCGATGAG | Amplification of *Mtb-fpg1* | This study |
| 7594 | GCCGTTGACGTTGACATACA | Amplification of *Mtb-fpg1* | This study |
| 7595 | CCTACGCTACGTGGTGGATT | Amplification of *Mtb-sigA* | This study |
| 7596 | TGGATTTCCAGCACCTTCTC | Amplification of *Mtb-sigA* | This study |
| 9257 | ATGGTCTCGGATCACCAAGT | Amplification of *Mtb-nei2* | This study |
| 9258 | CATCAACGACACCAGATCGT | Amplification of *Mtb-nei2* | This study |
| 4248 | GCGCCGCACCACCTCGACTT | Amplification of repeat region | (Spurgie*sz et a*l., 2003) |
| 4249 | CCGGGCAAAACCTCCGCCTAAC | Amplification of repeat region | (Spurgie*sz et a*l., 2003) |
| Restriction enzymes employed: NdeI, XhoI, NotI; cut sites underlined | | | |
| **Substrates for enzyme assays** |  |  |  |
| N248 | GGCGGCATGACCC**8oxoG**GAGGCCCATC | ^32^P labelled 8-oxo-7,8-dihydroguanine (8oxoG) | Eurogen- tec, Belgium |
| 2117 | GGCGGCATGACCC**U**GAGGCCCATC | ^32^P labelled uracil (U) | Eurogen- tec, Belgium |
| T248 | GATGGGCCTC**C**GGGTCATGCCGCC | Complementary strand to N248 | Eurogen- tec, Belgium |
| 1393 | GATGGGCCTC**A**GGGTCATGCCGCC | Complementary strand to N248 | Eurogen- tec, Belgium |
| 1394 | GATGGGCCT**C**GGGGTCATGCCGCC | Complementary strand to N248, 2117 | Eurogen- tec, Belgium |
| 1395 | GATGGGCCTC**T**GGGTCATGCCGCC | Complementary strand to N248 | Eurogen- tec, Belgium |
| 2798 | GCATGCCTGCACGG**5OHC**CATGGCCAGATCCCCGGGTACCGAG | ^32^P labelled 5-hydroxy cytosine (5OHC) | Eurogen- tec, Belgium |
| 2797 | GCATGCCTGCACGG**5OHU**CATGGCCAGATCCCCGGGTACCGAG | ^32^P labelled 5-hydroxy uracil (5OHU) | Eurogen- tec, Belgium |
| 2796 | GCATGCCTGCACGG**diHU**CATGGCCAGATCCCCGGGTACCGAG | ^32^P labelled dihydroxy uracil (diHU) | Eurogen- tec, Belgium |
| 2873 | CTCGGTACCCGGGGATCTGGCCATGGCCGTGCAGGCATGC | Complementary strand to 2798, 2797, 2796 | Eurogen- tec, Belgium |

**Table S3. Substrate specificity of *M. tuberculosis*** Mtb-Fpg1.

| **Substrate** | **Mtb-Fpg1 ^†^** |
| --- | --- |
| faPy | +++ |
| 8oxoG*:C | +++ |
| 8oxoG*:T | +++ |
| 8oxoG*:A | - |
| 8oxoG*:G | +++ |
| 5OHC*:G | + |
| 5OHU*:G | - |
| diHU*:G | - |
| U*:A | - |
| U*:G | - |
| A*:8oxoG | - |
| Alkylated bases | - |

* denotes P^32^-labelled strand

**^†^** +++ denotes very high efficiency on a substrate, + good efficiency on a substrate,

- complete lack of activity on a substrate

**Table S4. Overview of characterization of mycobacterial Fpg/Nei orthologs to date.**

| **Species** | **Fpg/Nei orthologs** | **Analysis** | **Reference** |
| --- | --- | --- | --- |
| *M. tuberculosis* H37Rv | Mtb-Fpg1  (Rv2924c) | - Purified protein shows high 8oxoG and met-faPy activity and low 5-hydroxycytosine activity - Purified protein show no activity towards oxidized pyrimidines, uracil, 5-hydroxyuracil, dihydrouracil, and alkylated bases - Gene expression levels assessed in *M. tuberculosis* vary depending on different lengths of the tandem repeats upstream of *Mtb-fpg1* (VNTR3239) | This study |
| *M. tuberculosis* 36KAZ | *Mtu*-Nei2  (Rv2464c homolog) | - Complementation of *E. coli*: Decreases the mutation frequency of an *E. coli fpg mutY* strain - Purified protein shows substrate specificity that more closely resembles *E. coli* Nei than *E. coli* Fpg, but also unique qualities | (Sidoren*ko et a*l., 2008) |
| *M. tuberculosis* KHA94 | *Mtu*-Fpg2  (Rv0944c homolog) | - Complementation of *E. coli*: Moderately increases the mutation frequency of an *E. coli fpg mutY* strain | (Sidoren*ko et a*l., 2008) |
| *M. tuberculosis* | Rv2924c | - Complementation of *M. smegmatis*: Complements *M. smegmatis* 8oxoG activity in cell-free extracts - Restores survival/ complements *M. smegmatis fpg* in H_2_O_2_ survival assay | (Ja*in et a*l., 2007) |
| *M. smegmatis* mc^2^155 | MsmFpg  (Rv2924c ortholog) | - Cell-free extracts show 8oxoG activity - *M. smegmatis* mutant is more sensitive to H_2_O_2_ than wt *M. smegmatis* and wt *M. tuberculosis.* Fpg complemented strains - *M. smegmatis* mutant shows higher mutation frequency than wt - *M. smegmatis* mutant shows different mutation spectrum than *E. coli fpg* mutant - *M. smegmatis* mutant shows preferential incorporation of G opposite 8oxoG in cell-free extracts | (Ja*in et a*l., 2007) |

**Figure S1. Predicted domains and motifs in Fpg/Nei homologs.** Multiple Fpg/Nei homologs of *Mycobacterium tuberculosis* depicted with the N-termini on the left and the C-termini on the right. Each enzyme contains a helix-two-turn-helix (H2TH) domain and zinc(Zn) finger motif. However, the catalytic domain is absent in Rv0944.

catalytic

H2TH

Zn

*Mtb*-Fpg-1

Rv0944

H2TH

Zn

Rv2464c

H2TH

catalytic

Zn

Rv3297

H2TH

catalytic

Zn

Fig. S1

**References:**

[1] Alseth I, Eide L, Pirovano M, Rognes T, Seeberg E & Bjørås M (1999) The *Saccharomyces cerevisiae* homologues of endonuclease III from *Escherichia coli*, Ntg1 and Ntg2, are both required for efficient repair of spontaneous and induced oxidative DNA damage in yeast. *Mol.Cell Biol.* **19**: 3779-3787.

[2] Jain R, Kumar P & Varshney U (2007) A distinct role of formamidopyrimidine DNA glycosylase (MutM) in down-regulation of accumulation of G, C mutations and protection against oxidative stress in mycobacteria. *DNA Repair (Amst)* **6**: 1774-1785.

[3] Sidorenko VS, Rot MA, Filipenko ML, Nevinsky GA & Zharkov DO (2008) Novel DNA Glycosylases from Mycobacterium tuberculosis. *Biochemistry (Mosc)* **73**: 442-450.

[4] Spurgiesz RS, Quitugua TN, Smith KL, Schupp J, Palmer EG, Cox RA & Keim P (2003) Molecular typing of *Mycobacterium tuberculosis* by using nine novel variable-number tandem repeats across the Beijing family and low-copy-number IS6110 isolates. *J.Clin.Microbiol.* **41**: 4224-4230.
